# Tracking implementation and (un)intended consequences: a process evaluation of an innovative peripheral health facility financing mechanism in Kenya

**DOI:** 10.1093/heapol/czv030

**Published:** 2015-04-28

**Authors:** Evelyn Waweru, Catherine Goodman, Sarah Kedenge, Benjamin Tsofa, Sassy Molyneux

**Affiliations:** ^1^Department of Public Health Research, Kenya Medical Research Institute—Wellcome Trust Research Programme, P.O. Box 230, 80108, Kilifi, Kenya,; ^2^Department for Global Health and Development, London School of Hygiene & Tropical Medicine, Keppel St., London, UK,; ^3^Department of Monitoring and Evaluation - Referral System Strengthening, MEASURE Evaluation/ICF International, Nairobi, Kenya and; ^4^Centre for Tropical Medicine, Nuffield Department of Clinical Medicine, University of Oxford, Oxford OX3 7LJ, UK

**Keywords:** Accountability, Africa, community involvement, decentralization, peripheral facility financing, quality of care, relationships, user fee removal

## Abstract

In many African countries, user fees have failed to achieve intended access and quality of care improvements. Subsequent user fee reduction or elimination policies have often been poorly planned, without alternative sources of income for facilities. We describe early implementation of an innovative national health financing intervention in Kenya; the health sector services fund (HSSF). In HSSF, central funds are credited directly into a facility’s bank account quarterly, and facility funds are managed by health facility management committees (HFMCs) including community representatives. HSSF is therefore a finance mechanism with potential to increase access to funds for peripheral facilities, support user fee reduction and improve equity in access. We conducted a process evaluation of HSSF implementation based on a theory of change underpinning the intervention. Methods included interviews at national, district and facility levels, facility record reviews, a structured exit survey and a document review. We found impressive achievements: HSSF funds were reaching facilities; funds were being overseen and used in a way that strengthened transparency and community involvement; and health workers’ motivation and patient satisfaction improved. Challenges or unintended outcomes included: complex and centralized accounting requirements undermining efficiency; interactions between HSSF and user fees leading to difficulties in accessing crucial user fee funds; and some relationship problems between key players. Although user fees charged had not increased, national reduction policies were still not being adhered to. Finance mechanisms can have a strong positive impact on peripheral facilities, and HFMCs can play a valuable role in managing facilities. Although fiduciary oversight is essential, mechanisms should allow for local decision-making and ensure that unmanageable paperwork is avoided. There are also limits to what can be achieved with relatively small funds in contexts of enormous need. Process evaluations tracking (un)intended consequences of interventions can contribute to regional financing and decentralization debates.

**Key Messages**

- Alternative financing mechanisms to user fees are needed in peripheral facilities.
- Health sector services fund (HSSF) is an innovative direct facility funding mechanism in Kenya.
- Early national experience with HSSF suggests impressive achievements.
- Challenges include complex relationships and accountability requirements. Process evaluations are essential to track finance interventions.

## Introduction

Peripheral public health facilities play a potentially valuable role in the implementation of primary health care in developing countries, but face significant challenges in levels of resources, quality of care and accessibility to potential users (Ranson *et al.* 2003). Such challenges, and pressure from funders, led many African countries to introduce user fees in the 1980s. Waivers and exemptions were incorporated into user fee policies to protect specific categories of patients from costs, such as young children, pregnant women and the poor (McPake *et al.* 2011; Ridde and Morestin 2011). User fees are now widely recognized as being inefficient in raising substantial revenues for health facilities (James *et al.* 2006). They have also been found to reduce demand for health services, especially among the poor. Intended improvements in access and quality of care have therefore generally not been realized (Lagarde and Palmer 2008; McPake *et al.* 2011; Ridde and Morestin 2011).

In response to user fee problems, many countries introduced user fee reduction or elimination policies in the 2000s, and new exemptions for particular patient groups or health conditions (Meessen *et al.* 2011). These policies have faced significant implementation challenges as they have often been introduced fast, without adequate planning and communication and without alternative sources of income for facilities. The result has been sudden increases in utilization of ill-prepared facilities, leading to concerns about quality of care. To prevent or overcome such challenges, facilities have often retained or re-introduced higher user fees (Meuwissen 2002; Lewis 2007; Chuma *et al*. 2009). Intended gains of user fee reduction or elimination have thereby been undermined.

Challenges with user fees and their reduction have contributed to interest in alternative peripheral facility finance mechanisms, including performance-based financing (PBF). Studies have shown that although PBF is a potentially powerful tool for increasing key targeted outputs, there can be perverse outcomes such as targets skewing activities and performance away from more complex services, increased administrative and monitoring burdens and costs, and gaming (Eldridge and Palmer 2009; Witter *et al.* 2012; Chimhutu *et al.* 2014). This literature suggests: (1) there is need for continued identification and tracking of a wider range of peripheral facility financing mechanism options; and (2) tracking of any facility finance intervention should not only consider if it works (black box approach), but also how it works or does not work, including with regards to interactions with the broader health system, how it is perceived by different stakeholders and the potential for unintended consequences [open box approach (Ssengooba *et al.* 2012; Borghi *et al.* 2013; Witter *et al.* 2013)]. The latter approach is relatively rare, but critical to contribute to knowledge about how to improve design and implementation of finance interventions (Witter *et al.* 2013).

Kenyan peripheral health facilities have faced many of the challenges related to user fee policy noted above and followed a similar policy journey. In Kenya, health centres and dispensaries control relatively few resources: the central Ministry of Health supplies facility infrastructure, qualified health staff, drugs and equipment; and provides money for operational expenses such as support staff, maintenance, allowances, fuel and non-medical supplies (MOPHS 1996). User fees were first introduced in the 1980s, together with district health management teams (DHMTs) and health facility management committees (HFMCs) (Oyaya and Rifkin 2003). HFMCs, including community representative members, became a requirement for all public health centres and dispensaries, with a role of overseeing general operations and management of facilities, including user fees.

In 2004, recognition that user fee charges were too high and variable led to the introduction of the ‘10/20’ policy. This policy aimed to reduce and standardize fees to 10 and 20 Kenyan Shillings (KSH) (∼0.15 US dollars and 0.29 US dollars) in dispensaries and health centres, respectively. Exemptions were mandated for all children under five, antenatal care and deliveries, and treatment for malaria, tuberculosis and sexually transmitted diseases. An implementation challenge was that central level funds for operational expenses were often failing to reach peripheral facilities. Funds cannot be spent at district level without an authority to incur expenditure (AIE), a fiscal receipt issued by Treasury through the Ministry of Health delegating financial authority and approving the expenditure of public funds according to an existing budget (Government of Kenya 2009b; Opwora *et al*. 2010). However, almost a third of allocations approved under AIEs were not received at district level in the mid-2000s (Ministry of Health 2007). In addition, facilities struggled to access funds distributed through districts due to bureaucratic and liquidity problems at the District Treasury which channels public funds for district activities, and most health sector funds were spent at DHMT level (Ministry of Health 2007). One approach to cope with the limited operational expenses was to continue to charge higher user fees. Thus facilities did not strictly adhere to the user fee reduction policy, and quality and access concerns remained (Opwora *et al*. 2010; Molyneux *et al*. 2012).

Recognition of these challenges contributed to the introduction of an innovative national health financing intervention called the health sector services fund (HSSF). HSSF goals for peripheral public health facilities are to increase resources for services, to account for these resources in an efficient and transparent manner, and to strengthen community involvement in facility management (Government of Kenya 2009a; Ramana *et al.* 2013).

Following a pilot in one province (Opwora *et al*. 2010), phased nationwide implementation began with public health centres in October 2010, and public dispensaries in July 2012. Under HSSF, the Government and development partners [mainly Danish international development agency (DANIDA) and the World Bank] contribute to a central fund, which is used to credit funds directly into an approved facility’s bank account every quarter. HSSF funds are intended to cover the facility’s operational expenses according to financial guidelines set out by the ministry for public health and sanitation (MOPHS) (MOPHS 2010), including items such as facility maintenance, refurbishment, support staff, allowances, communications, utilities, non-drug supplies, fuel and community-based activities. At facility level, HSSF funds are managed by HFMCs which have been given a stronger financial oversight role. HFMCs should include five residents of the facility catchment area with at least secondary school education, including three women, and four ex-officio members, including the health facility in-charge, and representatives of the provincial administration and the district medical officer of health (DMoH) (Government of Kenya 2009a). Other funds available to the facility, such as user fee revenue, and grants and donations received locally, should be banked in the same account, and be managed and accounted for together with HSSF funds. The Ministry continues to provide facility infrastructure, trained health workers, drug kits and medical supplies directly to facilities. It is recognized that HSSF alone will not be able to improve service delivery. However, HSSF has the potential to contribute to improved quality of care, adherence to user fee policies and ultimately improved equity in access to health care.

In this article, we present findings from an open box process evaluation of early experiences of HSSF in Kenya. The findings are relevant to ongoing discussions on the future of HSSF in Kenya, and have implications for international discussions on user fee reduction and removal, and alternative health financing options.

## Study methods

**Figure 1.**
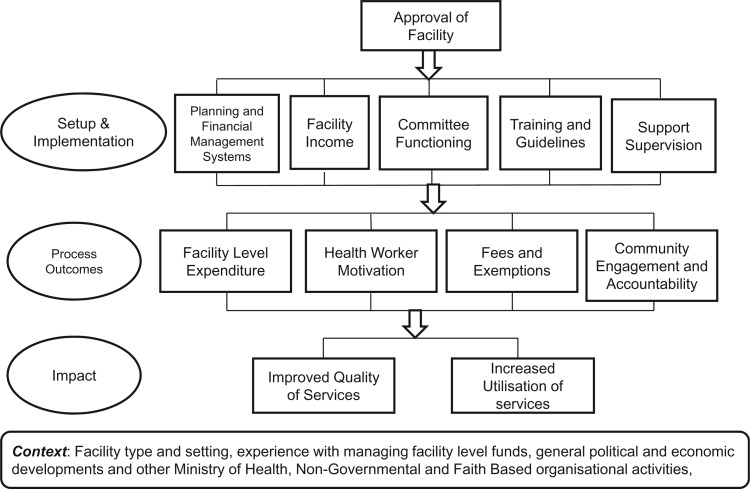
Theory of change underpinning HSSF. Source: (Opwora *et al*. 2010)

We conducted a process evaluation based on a theory of change underpinning the intervention (Figure 1

); a theory derived from the literature and discussions with stakeholders prior to national implementation (Opwora *et al*. 2010). The evaluation was conducted 1 year after national implementation in health centres, and ∼6 months after implementation in dispensaries. A case-control evaluation approach was not possible given nation-wide implementation in one stage, and it was considered too soon after implementation to include a quantitative pre–post comparison. Data collected included qualitative interviews at national, district and facility levels, facility record reviews, a structured exit survey and a document review. The approach and tools were based on the theory of change, but were kept deliberately open to allow for the identification of unintended and unexpected effects, including on the broader health system. Facility-based empirical work focused on health centres, given their longer experience with HSSF by the time of data collection, while national level interviews and document reviews covered HSSF in both health centres and dispensaries.

### National level key informant interviews

At the national level, we interviewed nine key informants representing the Ministry of Health, the HSSF Secretariat and international stakeholders from the World Bank, DANIDA and the Danish Embassy. Four key informants were interviewed in both 2012 and 2013, four only in 2012 and one only in 2013. Interviews concerned their roles in support, supervision and oversight of facility level funding, and their perceptions of HSSF and its implementation. Data on HSSF disbursements and expenditure patterns up to January 2013 were also obtained.

### District and facility level in-depth interviews

District and facility-based data collection took place in April and May 2012. Five districts were purposively selected to ensure a range of socio-economic levels and geographic locations (two rural, two urban and one mixed). In each district, one weak and one strong performing health centre were selected on the basis of discussions with DHMT members. Facilities perceived as substantial outliers in performance terms were excluded.

We interviewed DHMT and DMoH in each district, and five county-based accountants (CBAs). In the 10 selected health centres, we held individual interviews with the facility in-charge (total *n* = 10), and a focus group discussion with at least three HFMC members (10 discussions; 31 HFMC members). Interviews covered: HSSF training, materials and systems; user fees charged; changes in committees and community involvement in facilities; use of HSSF funds; financial management of HSSF funds; and the perceived impact of HSSF and factors influencing impact.

### Structured exit survey

In each of the 10 selected health centres, we interviewed 10 patients or their caretakers leaving the facility after receiving curative outpatient care (*n* = 99). Over half of these ‘exit interviewees’ were aged 25–44 years (60.6%), and about half were bringing a sick child for treatment (55.6%). Most (82.8%) were female, had completed primary school (84.9%) and reported literacy in Kiswahili (93.9%) and English (76.7%). The survey covered interviewees’ awareness of HFMC membership; any observed changes in the quality of care provided at the health facilities, and sources of information on HSSF.

### Facility record reviews

In all 5 districts and 10 health centres, we reviewed records on income and expenditure from January to December 2011. In the 10 facilities, we also observed whether information on income, expenditure, user fees, committee members, HSSF and patient rights was publicly displayed.

### Policy and programme documents

To supplement our own empirical work, we identified and reviewed policy and programme documents relevant to HSSF, including reports from an independent agency contracted to monitor on a quarterly basis HSSF’s fiduciary compliance, performance, governance arrangements and value for money (World Bank 2012). The fiduciary reports draw upon audits conducted in 20% of health facilities under HSSF, 50% of districts and all provincial and national level implementing entities. The authors received ethical approval from their institutions.

## Results

Following a brief overview of HSSF systems and procedures, we describe the process and experience of implementing HSSF in health centres and dispensaries in Kenya.

## Overview of HSSF systems and processes

HSSF resources should be credited directly to each designated facility’s bank account every quarter: 1339 USD for health centres, 327 USD for dispensaries and 1565 USD for DHMTs.

At the facility level, all funds including HSSF and user fees should be managed by the HFMC, which has responsibility for preparing and implementing the facility’s annual operational plan (AOP) and quarterly implementation plans (QIPs), and associated budgets. The planning process should be supported by a facility stakeholders’ forum organized by the facility in-charge, consisting of the HFMC, and local stakeholders including health development partners (Ramana *et al.* 2013). To account for funds received and used, facilities submit Monthly Expenditure Returns, Monthly Financial Reports and Quarterly Financial Reports using standard formats to the DHMT. These are just some of the financial management documents and reports that facilities have to fill for HSSF, as outlined in the Operational Guide (Table 1

**Table 1:** Documents required for the management of HSSF at facility level and their availability at the 10 health centres visited

Documents required at facility level, as specified in the ‘operational guide to the management of HSSF’	Total
	*N* = 10
Managing the HSSF—an operations guide	7
Guidelines on financial management for the HSSF	2
AOP	10
QIP	10
Chart of accounts	7
Memorandum vote book	8
Receipt book	4
Facility service register	3
Cash book	9
Cheque book	10
Cheque book register	6
Fixed assets register	9
Imprest register	7
Consumables stock register	8
Store register (stock cards)	10
Counter receipt book register	7
Receipt vouchers (F017)	5
Payment vouchers (F021)	10
Safari imprest form (F022)	5
Local purchase orders (LPO)	6
Local service orders (LSO)	4
LPO register	4
Request for quotations (RQF)	6
Stock cards (drugs) for all items in stores	10
Imprest warrants	6
Imprest register	6
Bank reconciliation forms (F030)	7
Counter requisition and issue vouchers (S11)	8
Counter receipt vouchers (S13)	10
Handover forms	3
Monthly service delivery report forms (MOH105)	9
Monthly financial report forms (MFR)	5
Monthly expenditure report forms (MER)	5
Quarterly financial report forms (QFR)	4
Outstanding imprest file	6
Bank statements file	10

Sources: in-charge interviews and facility record reviews.

) (Government of Kenya 2009a).

The DHMT review and monitor facility records and reports, and account for their own funds in similar ways to facilities. The DHMT submits a consolidated monthly financial report to MOPHS at the national level, through a HSSF secretariat responsible for overall management and oversight of the fund. The District Treasury was not given a specific role in HSSF, although it does have general fiduciary oversight of all government activities in the district, including health facilities. When reports are approved, money for the next quarter is transferred directly into the facility and DHMT bank accounts from national level, and AIEs are issued for each facility at national level.

DHMT HSSF funds are primarily to cover supportive supervision of facilities. In addition, CBAs were employed to offer additional ‘hand-holding’ in HSSF financial management to peripheral facility and DHMT staff, and to provide a link between facilities, districts and the HSSF secretariat. In mid-2013, there were 100 CBAs employed (an average of 2 per county). Should further financial management support be needed by facilities, they can use HSSF funds to contract accounts clerks.

Prior to HSSF roll out in 2010; the HSSF secretariat facilitated a 5 day training course for provincial managers, who in turn organized 4–5 day training workshops in their respective districts for facility in-charges and 2–3 HFMC members per facility. In addition to HSSF modalities, the training covered the use of Electronic Tax Register machines which were introduced in facilities in early 2011 to keep track of patient user fee payments. To support training, two HSSF manuals were produced, and later updated.

### Accessing, planning, managing and reporting on HSSF funds

22 841 758 USD had been allocated to HSSF by January 2013, with the largest contributors being DANIDA (44%), the World Bank (42%) and the Government of Kenya (14%). The total amount disbursed was 21 406 580 USD, with the highest proportion disbursed to health centres (52%), followed by dispensaries (22%) and DHMTs (21%); 4% went to the HSSF secretariat. By January 2013, funds were being sent to 262 DHMTs, 751 health centres and 2296 dispensaries. For financial year 2012/13, HSSF funds represented only 0.9% of the total ‘on-budget’ funding for the Kenyan health sector (Republic of Kenya 2012; World Bank 2012).

### AOPs, QIPs and AIEs

All DHMTs and facilities audited in the quarter ending September 2012 were operating bank accounts for HSSF funds (World Bank 2012), but only about half (56% DHMTs and 46% facilities) had received HSSF funds by the start of the quarter. Over the same period, most DHMTs and facilities (92 and 89%, respectively) had received the AIEs on time. These figures had been improving over time.

In our interviews in districts and health centres, there were generally positive responses regarding having received HSSF funds and structured AIEs. Furthermore, having AIEs that also covered user fees reportedly assisted with transparency, and in reducing conflicts between in-charges and HFMCs about allocation of user fee revenues. However, concerns raised included delays in accessing funds and receiving AIEs, AIEs not reflecting QIPs (particularly in rural areas), and how to spend money allocated in AIEs to items not useful for facilities. Delays were attributed to facility AIEs having to be signed off quarterly in Nairobi, and facilities having to wait for others in the district before QIPs or monthly reports were forwarded to the national level by DHMTs.

There is a straightjacket on HSSF because they had given us sort of an AIE…it has to be utilized on the item which is indicated… even if there is a shortage you can’t supply or you can’t provide the service out of that context (HFMC member).

Delays in funds and AIEs remained a major concern given the potential to undermine a key HSSF goal—to reduce the complexity and delays in accessing funds for facilities. Given that user fees were banked and accessed with HSSF funds, these delays were also impacting on access to user fees. Delays therefore reportedly brought ‘everything to a standstill’.

### Financial reporting and documentation

Fiduciary reports show that most audited facilities (79%) had not prepared the Monthly Financial Reports or Quarterly Financial Reports for the quarter ending September 2012. On the basis of selected key performance indicators related to reporting and documentation, 22% of facilities were rated satisfactory, 62% average and 16% poor, although these figures had been improving every quarter; an improvement also noted for DHMTs.

Many interviewees reported that completion of required reports took significant amounts of in-charges’ time, with one national level interviewee estimating that in 2013 in-charges were spending 20% of their time on accounts. Balancing the time requirements for accounting, documentation and patient care was described as extremely difficult:

Heh!… That [paperwork] is one of the most challenging things in HSSF; one thing I cannot say I’m 100% sure how they are supposed to be done. But I believe somehow I’m trying … and I have signed a performance contract with the government, so I’m [still] supposed to see maybe per day 30 clients you see. Now balancing the two it’s a challenge (In-charge).

District and facility managers relied heavily on CBAs to assist with financial accounting and documentation. CBAs assisted facilities in prioritizing among the many required financial management documents, and improvising to cope with the frequent unavailability of official versions of documents (Table 1). Facilities for example make their own ledgers and forms using photocopies or standard black exercise books.

## The role of districts and CBAs in overseeing HSSF funds

DHMT members reported some lack of clarity between the DHMT and CBAs regarding responsibility for conducting and funding the day to day training and support for in-charges on financial management. Furthermore, it became apparent that CBAs were reporting directly to the HSSF secretariat accountant, and ‘bypassing’ the DHMT, rather than working through it, as had initially been intended. The importance of embedding CBAs within DHMTs was regularly commented upon in interviews. Concerns may have been linked to some lack of support from DHMTs: HSSF led to their losing control over facility funds which had previously been channeled through them; they were being directly allocated relatively small amounts:

The requests from [DHMT] departments are just overwhelming and the amount is so small. You don’t know who/how to allocate that money to; it is so little (DHMT member).

A specific concern for some was the lack of involvement of the District Treasury in HSSF systems and procedures. As noted in a 2013 Aide Memoire:

Posting of accountants to districts is yet to help improve compliance with the government of Kenya fiduciary procedures under HSSF. They are not linked with the District Treasury for technical support and integration within DHMTs remains weak and accountants are not always included during supervision visits to facilities (DANIDA–World Bank, 2013).

Many interviewees felt that these ways of working were contributing to the verticalization and centralization of HSSF. However, whether or not this was a major concern was a source of debate. For example, one national level interviewee described the lack of involvement of the District Treasury as having been ‘the Achilles heel’ of HSSF, while another argued that the establishment of a parallel system had been necessary precisely because of the failings of the District Treasury; and that integration with Treasury would essentially lead to a collapse of HSSF. Those against integration also noted that this verticalization and parallel system was a general problem for the Ministries of Health, and not an issue specific to HSSF.

### HFMCs and facility oversight

The Fiduciary Report for the quarter ending September 2012 found that all health facilities visited had a properly constituted HFMC, and that most (92%) had met at least once during the quarter to discuss the operations and activities of the respective facilities.

These nationally representative findings were reflected in the 10 health centres we visited. All had active committees, most of which were re-constituted within the last 3 years, in accordance with the guidelines. In addition to meeting at least every quarter, most facilities also reported monthly meetings for executive committee members. HFMCs, and particularly secretaries and chairmen, were generally reported to be committed to their duties, which they primarily described as linking the community to the health centre. Challenges reported included the need for more training on modalities of HSSF and their roles in its implementation, and some tension in relationships with in-charges. Four in-charges reported being uncomfortable with the constant questioning or ‘interrogation’ from HFMCs:

Some of them are not well oriented in their roles and responsibilities, most of them were, but for those who were not you’ll find that they have overstepped their mandate…this is in terms of financial management they act like auditors but not overseers of the implementation,… that has been a big problem because now it brings a bad line between the facility in-charge and the committee (DHMT member).

HFMC members were reportedly only receiving allowances of KSH 500/= (6 USD) per quarter, as standardized across the country, for attending quarterly meetings (maximum four per year). These payments were considered inadequate (‘peanuts’), even for an essentially voluntary role, given the amount of time involved, and were described as demotivating particularly for urban HFMCs.

Of interest is that only 38.8 and 18.0% of rural and urban exit interviewees, respectively, had heard of HFMCs. Only 37.4% of all exit interviewees had heard of HSSF; of these, most had heard about it from the radio (25.6%) or health facility (23.3%). Only 16.2% of those who had heard of HSSF described it correctly.

### Facility income and expenditure

None of the 10 facilities where information on facility income was collected reported adhering to the user fee policy at the time of free care for under fives and KSH 20 for over fives (Table 2

**Table 2.** User fees reported at 10 health centres visited (USD using 2011 average conversion rate 1 USD = 84 KSH)

User fees charged for	Expected charges	Rural health centres			Urban health centres			Total		
		Mean	Median	Range	Mean	Median	Range	Mean	Median	Range
2-year-old with malaria	0	0.24	0.24	0–0.6	0.21	0	0–0.83	0.23	0.12	0–0.83
Adult with malaria	0	1.45	1.67	0.47–2.38	0.81	0.83	0.24–1.67	1.13	0.95	0.24–2.38
2-year-old with pneumonia	0	0.12	0	0–0.36	0.19	0.24	0–0.48	0.15	0.12	0–0.48
Adult with pneumonia	0.24	0.98	1.07	0.48–1.43	0.45	0.24	0.24–1.07	0.71	0.60	0.24–1.43
Adult with TB	0	0.14	0	0–0.71	0.31	0.24	0–0.83	0.23	0	0–0.83
Adult with gonorrhoea	0	2.02	1.19	1.07–3.45	1.29	0.83	0.24–3.45	1.65	1.19	0.24–3.45
Woman at first ANC visit	0	2.64	2.98	1.43–3.81	0.98	1.19	0.24–2.02	1.81	1.43	0.24–3.81
Mother delivering	0	5.71	5.95	3.57–7.14	1.24	0.24	0–3.57	3.48	3.57	0–7.14

Source: in-charge interviews.

), although some did exempt children with malaria (most urban facilities), children with pneumonia (most rural) and adults with Tuberculosis (TB) (most rural). The total income from user fees for facilities as derived from facility records for 1 year (January to December 2011) ranged from 910 to 25 455 USD, while the equivalent figures for HSSF funds were 3870 to 6543 USD (Table 3

**Table 3.** Income from user fees and HSSF funds in 2011 at 10 health centres visited (USD)

	Rural health centres			Urban health centres			Total		
Income source	Mean	Median	Range	Mean	Median	Range	Mean	Median	Range
User fees	7065	3348	910–14 854	6476	2007	972–25 455	6770	2502	910–25 455
HSSF	4864	5208	3869–5208	5505	5214	5202–6542	5185	5208	3869–6542
Other*	0	0	0	1862	0	0–9312	931	0	0–9312

*Income from other sources includes output-based aid for specific services in maternal and child health, and financial donations, both of which were only recorded in one facility.

Source: facility record review.

). User fees and HSSF funds each represented approximately half of total facility income, with the proportion from HSSF in each facility ranging from 17 to 85%.

**Figure 2.**
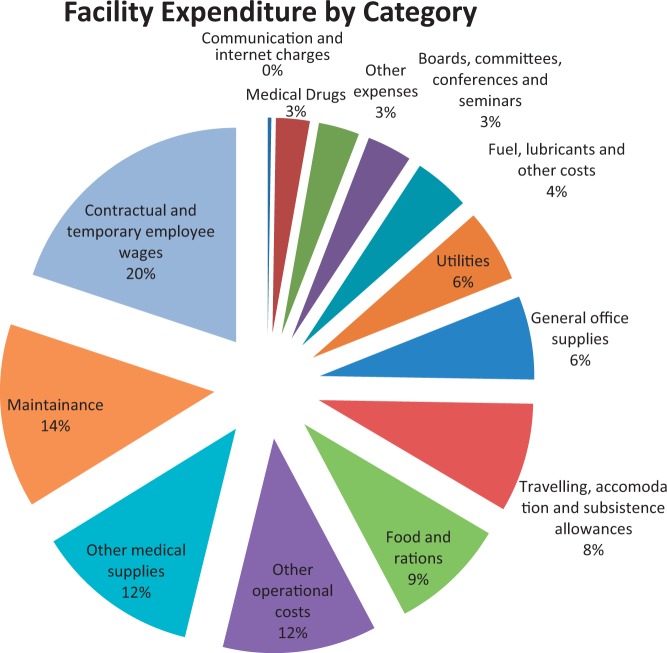
Use of HSSF funds by all facilities receiving HSSF between July 2011 and December 2012. Source: HSSF secretariat, May 2013

Expenditure figures from national records of all facilities receiving HSSF (Figure 2

) show that almost a quarter of facility funds were spent on wages, primarily for accounts clerks, watchmen/security staff, groundsmen and cleaners. High proportions of funds were also allocated to medical supplies (14%), travelling accommodation and subsistence (13%), other operational costs (11%), fuel and lubricants (6%) and maintenance (6%). Only 2% of funds were reportedly spent on purchasing drugs. In qualitative interviews, urban health centres reported water, sanitation (toilets and cleaners) and minor renovations as their most important uses of HSSF funds, while rural facilities named casual labourers, essential drugs, food and referrals. HFMC members also mentioned the importance of HSSF in paying their allowances for meetings. Interviews revealed some lack of clarity on whether HSSF funds could be used to buy drugs or hire accounting clerks.

Some expenses that were rejected by the DHMT, CBA or HSSF secretariat included newspapers, transport other than designated ambulances for referrals, major renovations, locum health workers and furniture. These became known as ‘ineligible expenses’ during initial tranches, and members of the HSSF secretariat stated that they would be increasingly heavily sanctioned where noted. Responsibility for ineligible expenditure ultimately rests with government employees; that is the in-charge and the DMOH as opposed to HFMC members. Potential consequences are known to include demotion, suspension, transfer, sacking and salary deductions, with these consequences described as contributing to some major anxiety and inaction among some staff. Several in-charges were reportedly ‘kwamad [blocked] by terror’ at the thought of having to lose their salary through accounting errors, preferring not to use or report on funds at all than risk such sanctions.

Beyond ineligible expenditure, there were some cases of misuse of funds such as user fee money being pocketed rather than recorded, a HFMC member taking a cash advance and then claiming to have been robbed, and an in-charge trying to forge signatures of HFMC members and transfer money to a personal account. However, none of these cases were from the 10 health centres we visited, and in national interviews, we were informed that only 10 or so people throughout the country (primarily DMOHs) had suffered salary deductions for expenses that could not be explained.

## Impact of HSSF funds on facilities’ quality of care and utilization

Interviews and fiduciary reports suggested that HSSF funds were generally being well used, with facilities able to improve their upkeep, buy consumables to improve quality of care, and ensure visible improvements in facilities and service delivery (World Bank 2012; Ramana *et al.* 2013).

Of course [quality of care] has improved; initially if you didn’t have gloves you would tell a client “we are sorry, we can’t help you” (In-charge).

Paying bills we are able to, before we used to get a backlog of bills but now we are able to pay our bills in time… we are able to even to collect drugs if for example we are out of stock; we can fuel a vehicle to go to the rural facilities and transport whatever excess drugs they have to our facility and make use of them. So it has made many things possible (In-charge).

Improvements have reportedly been particularly visible and impressive in dispensaries, where HSSF was described in national interviews as a ‘huge success’.

More than three-quarters (78.6%) of exit interviewees reported an improvement in overall service delivery at the facility over the previous year (and only 2.2% a deterioration). Approximately two-thirds reported improvements in facility cleanliness (60.6%), waiting time (59.6%), treatment given (59.6%), and about half improvements in the availability of medicine (50.6%), and the number and courtesy of staff (44.9 and 43.8%, respectively). In-charges said there were more patients coming to the facility because of the availability of drugs and lab reagents for testing, the improved general condition of the facility, increased outreach programmes and affordable prices relative to private clinics. In one case, HFMC members attributed the increased utilization to reduced user fees, which they introduced as a result of HSSF funds. However, this reduction in user fees was only reported in one facility; with most facilities maintaining user fees well above official levels as noted above.

Within this overall positive picture, challenges alluded to throughout the above results clearly had negative implications for quality of care and motivation of facility and management staff. These included the amount of paperwork, complaints about inadequate levels of funds at facility and district level, and debates about roles and functioning of committees, districts and CBAs. Cross-cutting all of these concerns was a sense by some in-charges of being over-monitored; one mentioned that there was a quarter where the facility had undergone so many external audits that health workers had felt ‘harassed’.

## Discussion

There are important limits to our study design: we cannot generalize from our 10 purposively selected health facilities; we cannot quantitatively compare data from those facilities with an appropriate baseline or control group; and we did not measure technical quality of care. However, a number of important conclusions can be drawn. Overall, experience with HSSF suggests that peripheral finance mechanisms can have important positive impacts on facilities in terms of ensuring that funds reach facilities, and that such funds can be overseen and used in a way that strengthens transparency and community involvement. HFMCs, one of the most widely introduced community accountability mechanisms across sub-Saharan Africa (Molyneux *et al*. 2012), can play an important role in that success, although with some limitations with regards to clarity of role and downwards accountability to communities. Our interviews suggest that having even relatively small amounts of funds being spent on casual staff, basic improvements of facilities and simple day to day facility needs can have an important impact on the condition of facilities, health workers’ motivation, patient satisfaction and ultimately on quality of care and utilization. Experience with HSSF also illustrates the possible positive impacts of a new finance mechanism on the wider health system. For example, the application of HSSF financial accountability systems to user fees reportedly strengthened community involvement in decision making for all facility funds, and reduced disputes between community representatives and frontline providers. These positive impacts were achieved through a finance mechanism in which funds are allocated across facilities based only on facility type, without any additional funding based on performance on key indicators. This observation, also made for an in-depth post-hoc evaluation of the pilot of this initiative on the Coast (Opwora *et al*. 2010), is important given the growing interest in—but heated debate on—the appropriateness of performance-based funding as a means to align the incentives of health workers with public health goals in many developing country contexts.

Within this overall positive experience, our evaluation of HSSF implementation suggests a range of further issues for consideration in selecting and evaluating periphery facility financing mechanisms in the region.

### Balancing fiduciary oversight with administrative and monitoring burdens

One important challenge is the need to balance fiduciary oversight with administrative and monitoring burdens (Witter 2009). Given the potential for misappropriation and misuse in peripheral facilities (McPake *et al.* 2011), there is clearly a need for fiduciary oversight. However, HSSF experience illustrates the potential for these oversight mechanisms to undermine the initial intentions of a financing intervention. HSSF was introduced to ensure that peripheral facilities gain access to funds allocated to them by cutting the bureaucracy involved, and enabling them to use their funds efficiently and in a transparent manner. In practice AIE processing is centralized, takes time and—importantly—ties down not only HSSF funds but also what were previously relatively flexibly used user fees. Thus, there is a risk not only that gains from finance interventions are undermined, but also that there are broader unintended consequences. A change in HSSF after our study was completed was to shift from quarterly to annual AIEs. This may have reduced funding delays for facilities, but our data suggests that simplifying unnecessarily complex accounting procedures would also strengthen implementation.

Administrative and monitoring burdens were minimized in HSSF by CBAs, who played a key support role for in-charges, including through working with them to develop a range of coping strategies and some local decision-making space and flexibility within guidelines. It has been raised elsewhere that implementation ‘on the cheap’ should not be attempted (Ssengooba *et al.* 2012); in this case, there would be particular risks with removing or under-supporting such key support personnel, who essentially play an interface role. The literature suggests that staff who interface between actors within a complex system with different interests, relationships, modes of rationality and power (Helsinki 2001; Long 2001), have the potential to translate and re-shape interventions on the ground, and to contribute to feeding frontline priorities and concerns upwards through the system to change the design and implementation. We suggest this potential is recognized and harnessed.

### Clarifying roles and responsibilities and decision-making space at different levels

Clarity in the roles of stakeholders and implementers, and the nature of relationships between key actors, are recognized as critical to policy implementation (Bossert 1998; Conyers 2007). Beyond some concerns regarding relationships between HFMCs and in-charges (see also Waweru *et al*. 2013), and between DHMTs and CBAs, a specific area of discussion and debate in HSSF was the level of involvement of the District Treasury in HSSF, with views ranging from the lack of district involvement being ‘the Achilles heel’ of HSSF, to an argument that HSSF was purposively designed to be a parallel system—and at least initially—precisely to avoid the failings of the District Treasury.

**Figure 3.**
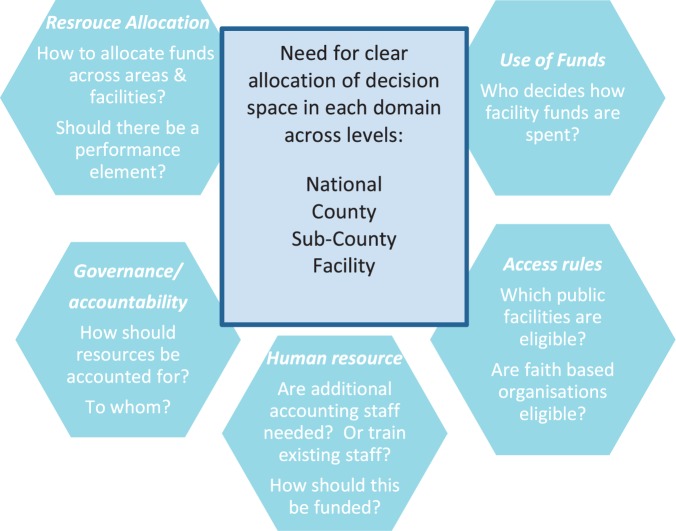
Allocating decision-space for HSSF across key domains for decision making

This verticalization/integration dilemma—whether or not to bypass or be integrated within systems that are perceived to be sub-optimally functioning—is likely to be faced in many peripheral facility finance interventions in the region. Although not easily resolved we suggest that it could be helpful to unpack finance interventions into a list of potential key decisions across several health system domains. For each decision, ideal ‘space’ at national, county/district and facility level can then be agreed and (re)negotiated over time. We suggest key decisions across key domains for HSSF in Figure 3

. Once decision-making space for key decisions is agreed, accountability systems downwards to communities and users, and upwards to managers and funders can be considered, including making sure that the total accounting responsibilities for each key actor are reasonable, and do not undermine their ability to achieve their roles (Waweru *et al*. 2013). Agreements clearly cannot be implemented until appropriate organizational structures and capacities are in place at each level. These issues and decisions are highly pertinent in Kenya today: the election of a new government in March 2013 initiated the implementation of a new constitution including devolution of government functions from national level to 47 semi-autonomous counties. The Kenyan devolution has important implications for government operations across all sectors, including health, in ways that continue to be debated at the time of writing.

### Interactions with user fees

We have noted above that peripheral finance mechanisms can have broader positive implications in facilities. Nevertheless, there are limitations to what a relatively small amount of fees can achieve. For HSSF, donors and the government had hoped funds would increase facility adherence to the 10/20 user fee policy, with positive implications for access especially for the poor (Opwora *et al*. 2010; Goodman *et al*. 2011; Molyneux *et al*. 2012). However, user fees did not reduce in health centres to the 20 shillings policy, nor were all exempted patients able to access care for free. In interviews it was repeatedly emphasized that HSSF funds, while greatly appreciated, were totally inadequate in meeting the diverse needs of the facility. User fees therefore remained a key source of income: these funds made up a substantial proportion of total facility funds, in some cases a higher proportion than HSSF funds. This finding supports those who have argued that user fee removal or reduction is far from a simple ‘stroke of the pen’ exercise (Gilson and McIntyre 2005), that additional resources to compensate for the loss of user fees are needed, and that funding allocations should be based on realistic assessments of facility needs.

Determining the appropriate level of compensation for user fee losses in facilities in Kenya is challenging, as compensating facilities according to current revenues will favour facilities with richer catchment areas and thereby exacerbate inequities. However, the 2013 presidential announcement that all user fees would be removed from peripheral facilities makes this task essential. Although it has been reported that HSSF funds have been increased for facilities to compensate for user fee losses, anecdotal evidence suggests that there is lack of clarity regarding this at facility level, and that many facilities are continuing to charge user fees, often with agreement from HFMCs and managers. The ways in which user fee removal has been implemented, and with what implications for quality of care, needs further exploration.

### The value of process evaluations

To develop a robust evidence base in this area, there is need to identify and track a wide range of peripheral facility financing mechanisms, including innovations such as HSSF. Our experience supports the importance of using in-depth open box process evaluations which consider if and how interventions work, interactions with the broader health system, stakeholders’ perceptions, and the potential for unexpected and unintended consequences. Witter *et al*. have recently published a valuable framework to support PBF studies, which can also be applied to other financing mechanisms (2013). Such frameworks, and in-depth process evaluations presented here, can assist in planning of appropriate future interventions.

## Conclusion

HSSF experience suggests that finance mechanisms can have a strong and broad positive impact on peripheral facilities. There have been impressive achievements in ensuring that funds reach facilities, and that all facility funds—including user fees—are being overseen and used in a way that strengthens transparency and community involvement. Also observed is that HFMCs, one of the most widely introduced community accountability mechanisms in sub-Saharan Africa, can play a valuable role in managing facilities. Challenges or unexpected outcomes suggest: complex and centralized accounting requirements can undermine efficiency goals of finance interventions; finance mechanism implementation problems can have wider negative impacts (in this case difficulties for facilities in accessing crucial user fee funds); and the need for clarity in the roles and responsibilities of key actors. There are also clearly limits in downwards accountability to users and communities, and to the possible achievements of one financing intervention in the context of wider challenges, including an unreliable drug supply, poor access to emergency transportation and shortages of qualified staff. Process evaluations tracking (un)intended consequences of HSSF and other similar interventions are needed to contribute to regional financing and decentralization debates.
